# Lipoic Acid Decorated Gold Nanoparticles and Their Application in the Detection of Lead Ions

**DOI:** 10.35248/2157-7439.19.10.539

**Published:** 2019-12-12

**Authors:** William Ghann, Tyler Harris, Daiyaan Kabir, Hyeonggon Kang, Mintesinot Jiru, Mohammed M Rahman, Meser M Ali, Jamal Uddin

**Affiliations:** 1 Center for Nanotechnology, Department of Natural Sciences, Coppin State University, 2500 W. North Ave, Baltimore, MD, USA; 2 Soil and Water Quality Analytical Lab, Department of Natural Sciences, Coppin State University, 2500 W. North Ave, Baltimore, MD, USA; 3 Chemistry department, King Abdulaziz University, Jeddah 21589, Saudi Arabia; 4 Department of Neurosurgery, Cellular and Molecular Imaging Laboratory, Henry Ford Hospital, Detroit, MI, USA

**Keywords:** Lead, Gold nanoparticles, Lipoic acid, Sensor, Colorimetric, Absorption

## Abstract

A simple colorimetric method has been developed for the detection of lead (Pb^2+^) in water samples using lipoic acid-functionalized gold nanoparticles. The lipoic acid-functionalized gold nanoparticles are induced to aggregate in the presence of the Pb^2+^ which results in a change in the color of the functionalized gold nanoparticles. The change in color and the amount of Pb^2+^ producing the change could be monitored via UV-visible spectrophotometry. A good correlation coefficient of 0.9927 was obtained for the calibration curve of the colorimetric method. The method was applied in the determination of Pb^2+^ in water samples and the results compared to that of measurement carried out with Atomic Absorption Spectroscopy.

## INTRODUCTION

Gold nanoparticles have attracted immense attention in recent years owing to their numerous application in biomedicine, electronics, catalysis, and biosensing [1–6]. The surface chemistry allows for attachments of drug and targeting agents that permits them to be used in targeted drug delivery [7]. Their large surface area also permits them to carry a large payload of cancer drugs to cancer sites in chemotherapeutic treatments [7]. Furthermore, the high density of the gold and its capacity to absorb X-rays makes it useful as an X-ray contrast agent [8]. One property of gold nanoparticles that makes them suitable for sensing applications is the Surface Plasmon Resonance (SPR) [6]. SPR occurs when electrons in the conduction band of a material simulated by an incident light oscillate collectively at the interface between negative and positive permittivity materials [9,10]. Thus, SPR makes the surface of gold nanoparticles very sensitive to external influence [11–13]. When other materials encounter the surface of the gold nanoparticles there is a shift in the plasmon band that can be monitored via UV-Vis spectrometry [14]. The shift occurs due to the change in the effective refractive index of the medium [15,16]. The change in wavelength could also be due to plasmon coupling between the closely packed particles which occurs when the particles aggregate. This phenomenon has been exploited in the sensing of heavy metals [17,18].

Lead is one of the most toxic metals found in the environment. Lead toxicity is an important environmental hazard and its effects on the human body are devastating. It is particularly detrimental for children as exposure to lead ion has been shown to cause brain and kidney abnormalities [19,20]. The presence of Pb^2+^ in water bodies continue to pose a serious threat to health and the environment. Several methods have been developed for detection of lead in the past [21–24]. The present work involves the use of gold nanoparticle-based sensors in the detection of Pb^2+^ in water samples. Some of the unique characteristics of gold nanoparticles are exploited for the detection of lead ion in water bodies. The unique characteristics of gold nanoparticles being taken advantage of include their small size, large surface area, ability to bind with other species and the surface plasmon. Gold nanoparticles functionalized with different kinds of ligands have previously been used in the detection of heavy metals [17,18]. The mechanism of detection depends on the sensitivity to change in size, aggregation state and refractive index of the gold nanoparticles upon attachment of metal ions [25,26].

In addition to heavy metals, gold nanoparticles have also been used in sensing different materials ranging from biomolecules to electronics [15,16]. Most recently, Ratnarathorn et al. [27] used maleic acid as a ligand on gold nanoparticles for the detection of lead. Maleic acid has two terminal carboxylic acid groups. One of the carboxylic acids in this case, was conjugated to the gold nanoparticle and the other to the lead ion. Nevertheless, the interaction between gold nanoparticles and carboxylic acids is not strong and thus the functionalized particles tend to be unstable. Ligands with thiol or sulfur terminal groups form strong bonds with gold nanoparticles and ensure the stability of the resulting nanoparticles.

## EXPERIMENTAL SECTION

### Techniques

Absorption spectroscopy was carried out with UV-3600 Plus from Shimadzu, MD, USA. Transmission Electron Microscopy (TEM) images were acquired on JEM-1400 PLUS (JEOL USA, Peabody, Massachusetts, USA). The images were viewed using Digital Micrograph software from GATAN (GATAN Inc., Pleasanton, CA, USA). For comparison purposes, the Pb content was also analyzed by Atomic Absorption Spectrometry (AAS) from PerkinElmer Corporation, Waltham, MA, USA). Dynamic Light Scattering (DLS) measurements were carried out using Horiba Particle Size Analyzer LB 550 from Horiba Instruments Inc., Irvine, CA, USA.

### Synthesis of gold nanoparticles

Synthesis of citrate-stabilized GNPs was carried out using a modified Frens method [28–30]. First, a 100 mL of 0.6 mM gold salt solution in a 250 mL Erlenmeyer flask equipped with a magnetic stirring bar was brought to boil on a hotplate. To this solution, 2 mL of 0.17 M sodium was added and the solution further stirred for another 15 minutes. A series of color changes, from light yellow to gray, to purple, and to ruby red was observed. The synthesized gold nanoparticles were characterized using the UV-Vis, DLS, and TEM.

### Ligand exchange reaction

The ligand exchange reaction was then carried out in order to replace citrate molecules with lipoic acid. The amount of lipoic acid used was based on the size of the gold nanoparticles as determined by dynamic light scattering measurements. Using the diameter of the gold nanoparticles, the surface of the gold nanoparticle and the number of ligands that could be attached to the surface of the gold nanoparticles were computed. Lipoic acid was used in excess to ensure complete exchange of the ligand. Specifically, 250 times excess of ligands was used to guarantee complete exchange of the citrate molecules. The reaction was also carried out under basic conditions (pH = 10) to ensure complete deprotonation of the carboxylic acid groups on the lipoic acid. To carry this out, 0.24 g of the lipoic acid was dissolved in basic water, added to the gold nanoparticles and allowed to stir at room temperature overnight. The particles were then purified by a series of centrifugation and the pellets dispersed in pure water for analysis.

## RESULT AND DISCUSSION

**Figure 1:**
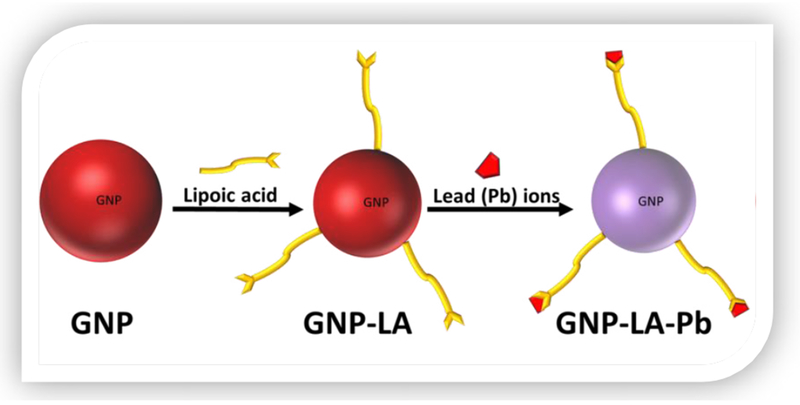
Mechanism behind colorimetric detection of Lead ions.

**Figure 2:**
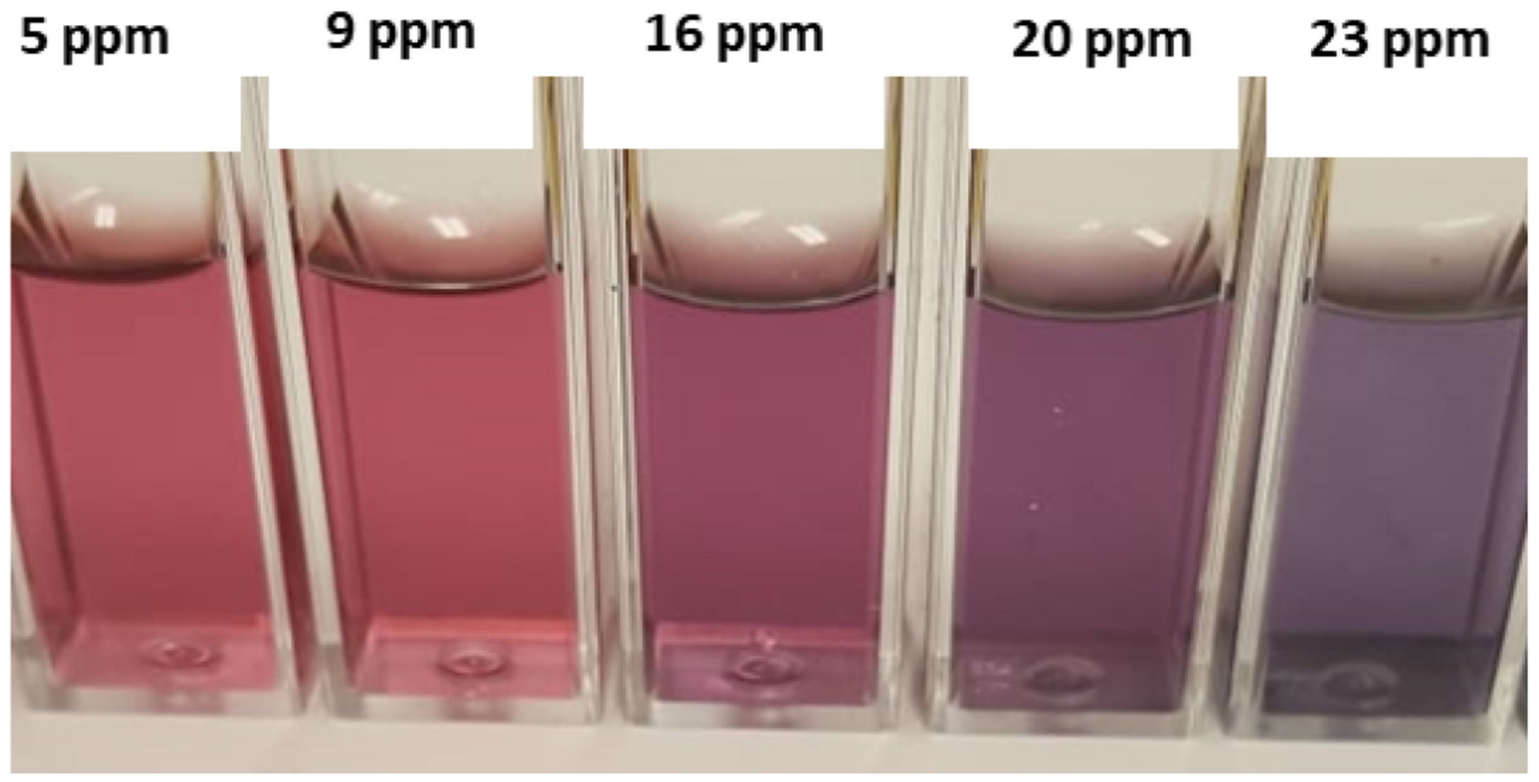
Gold Nanoparticle solution with different concentrations of lead ions.

In this study, the concentration of Pb ions in water samples was assessed by a colorimetric method using gold nanoparticles and the results compared with measurements carried out by Atomic Absorption Spectroscopy. Gold nanoparticles were first synthesized by the citrate method followed by a ligand exchange reaction to replace the citrate molecules as illustrated in Figure 1. The lipoic acid used as a ligand has a disulfide group at one end for attachment to the gold surface and a carboxylic acid at the other end for interaction with the Pb ions. Interaction with the Pb ion induces aggregations resulting in a change of color as displayed in Figure 2. It was observed that the ruby red color of the gold nanoparticles changes to a purple color upon the addition of lead ions to the gold nanoparticle solution.

### Dynamic light scattering studies

**Figure 3:**
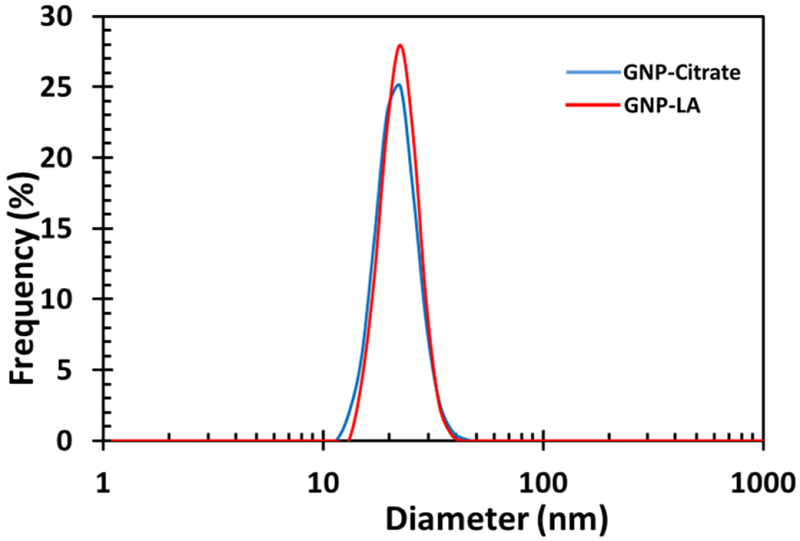
Dynamic Light Scattering measurement of gold nanoparticle before and after functionalization with lipoic acid.

The functionalized gold nanoparticles were characterized using dynamic light scattering (DLS) measurements. The DLS is a particle size analyzer that evaluates the size and size distribution of nanomaterials in solution. The size determined via DLS is usually bigger than that determined via Transmission Electron Microscope (TEM) imaging since DLS measures the hydrodynamic diameter which comprises of the particles itself and a kind of an electric dipole layer that sticks to the surface of particles in solution. TEM, on the other hand, measures the diameter of the exact image of the particles thus yielding more precise values. The DLS spectra of the citrate-stabilized gold nanoparticles (GNP-Citrate) and that of the corresponding lipoic acid coated gold nanoparticles (GNP-LA) are displayed in Figure 3. The average size of the citrate state stabilized gold nanoparticles were found to be 20.6 nm and that of the functionalized particles was 21.5 nm.

### Transmission electron microscopy imaging

**Figure 4:**
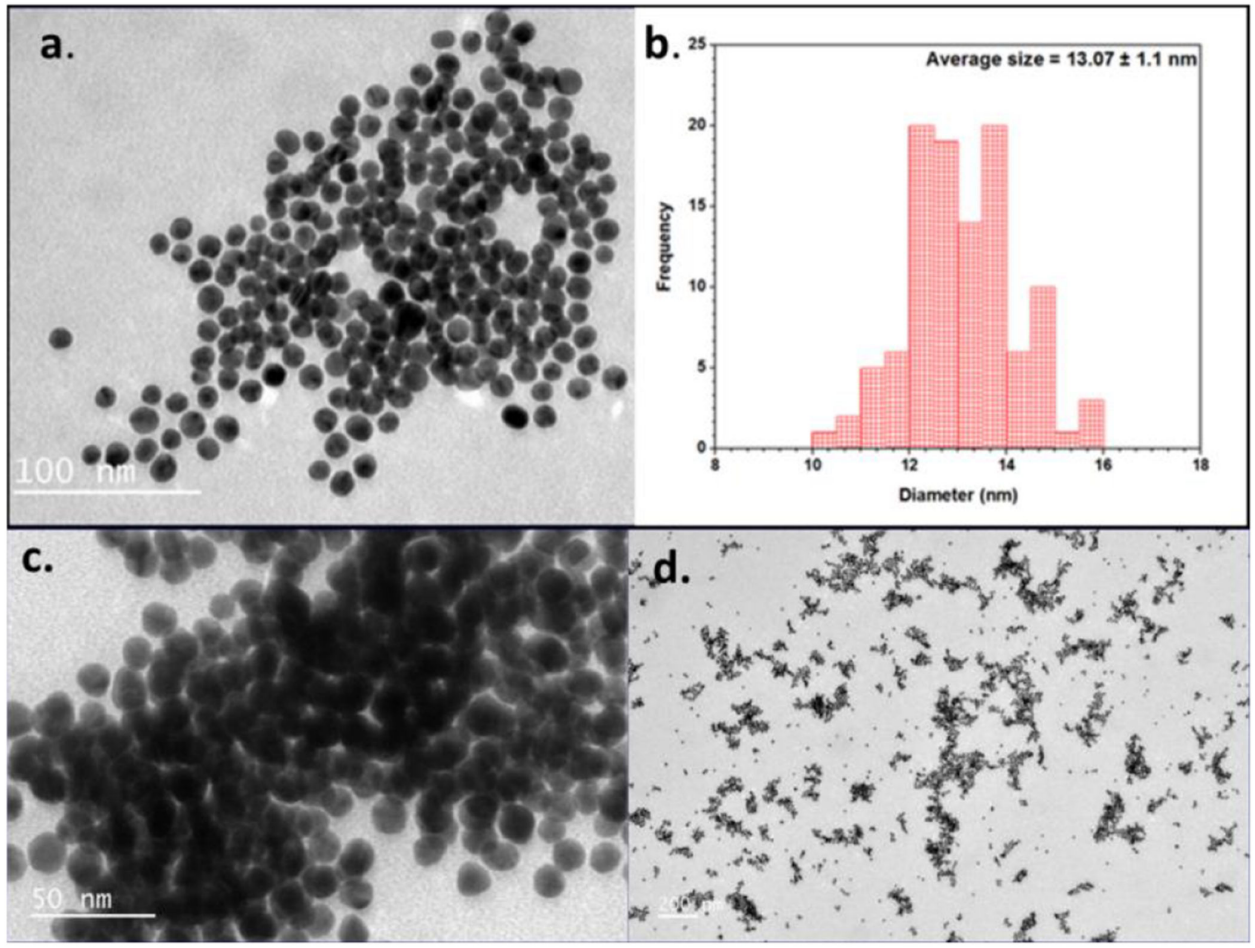
TEM image of gold nanoparticles before and after conjugation with Pb ions. (a) GNP-LA; b) corresponding histogram of GNP-LA-Pb; (c and d) GNP-LA-Pb at different magnifications.

The morphology of the lipoic acid conjugated gold nanoparticle (GNP-LA) was analyzed before and after interaction with lead ions using Transmission electron microscopy (TEM). The gold nanoparticles before conjugation with Pb ion show the particles to be uniformly dispersed with negligible aggregation. The TEM image of the lipoic acid conjugated gold nanoparticle along with the histogram of the size analysis is displayed in Figure 4. The uniformity of the size and shape of the lipoic acid conjugated gold nanoparticle before interaction with Pb ions is clearly demonstrated in Figure 4a. The average size of the functionalized gold nanoparticles as displayed in the histogram in Figure 4b is 13.10 nm. Figure 4c and 4d show the TEM images of the lipoic acid conjugated gold nanoparticles with the lead ions (GNP-LA-Pb). The average size of these particles was 13.90 nm which is about 0.8 nm bigger than the size of just lipoic acid functionalized gold nanoparticles. The increase in size suggests that the Pb^2+^ ions interacted with the carboxylic groups of the lipoic acid bound to the gold nanoparticles. It can also be observed in the TEM images that the gold nanoparticles with the Pb ions are more clustered, which indicates that the lead ions cause the gold nanoparticles to aggregate.

### UV-measurements

**Figure 5:**
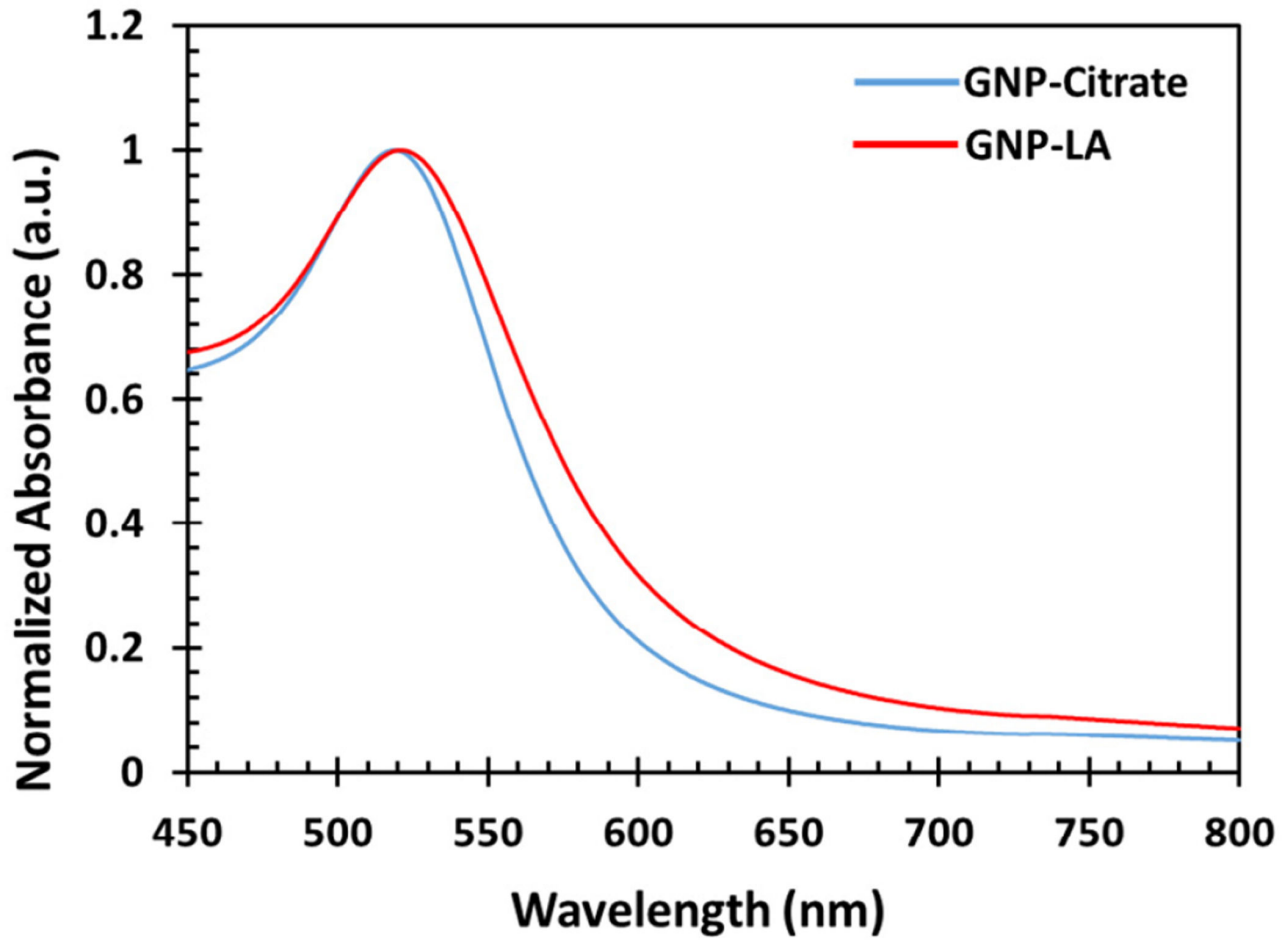
UV-Vis measurement of the lipoic acid functionalized gold nanoparticles before and after ligand exchange.

The synthesized gold nanoparticles before and after the ligand exchange reaction were further characterized by UV-Vis Spectroscopy. The characterization was carried out to assess the functionalization of the gold nanoparticles with the lipoic acid. Gold nanoparticles exhibit SPR, which is the collective oscillation of conduction band electrons in resonance with an incident light of a specific wavelength. The SPR of gold nanoparticles result in the absorption of light of wavelength between 500 nm and 600 nm depending on the size and shape of the nanoparticles. Thus, any changes on the surface of the gold nanoparticles results in the shift of the plasmon band, which can be monitored by UV-Vis Spectroscopy. An increase in size of the gold nanoparticle through aggregation or through binding with ligands results in a red shift of the Plasmon band. The UV-Vis spectra of the gold nanoparticles before and after the ligand exchange reaction is displayed in Figure 5. The surface plasmon band of the bare gold nanoparticles was 518 nm and this was red-shifted to 523 nm upon addition of the lipoic acid. A shift in the peak absorbance is indicative of a gold nanoparticle surface modification. In this case, it signifies the exchange of the lipoic acid with the citrate molecules.

**Figure 6:**
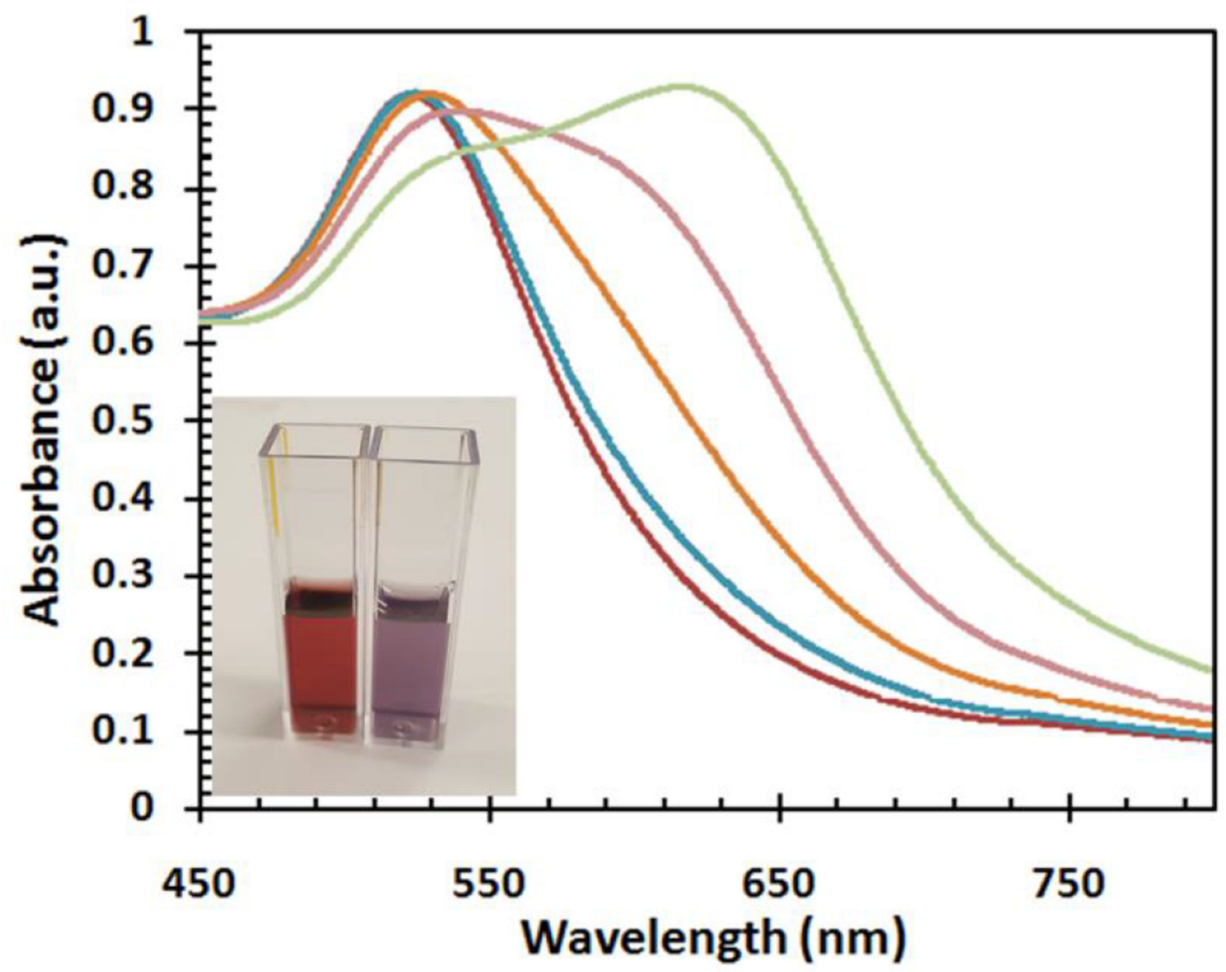
UV-Vis spectra of gold nanoparticle solutions with different concentration of lead ions.

To prepare a calibration for the colorimetric detection of lead ions using UV-Vis, a series of solutions with different concentration of lead ions were prepared. The lead solutions with concentration within the range of 1 to 20 ppm were added to the prepared lipoic acid coated gold nanoparticles and tested with UV-VIS spectrometer. Briefly, 1 mL of GNP-LA solution was added to 1 mL of a Pb^2+^ ion solution of a known concentration. The GNP-LA turned purple as they met Pb^2+^ at high ionic strength, indicating the formation of aggregates. Figure 6 shows the UV-Vis spectra of gold nanoparticle solutions with different concentrations of lead ions. It was observed that the higher the concentration of the lead ions the greater the degree of aggregation of the gold nanoparticles. The wavelength of the plasmon band in the absence of the lead ions is 523 nm but a red shift of plasmon band is observed with each increase in the concentration of the lead ions. Thus, a linear relationship was found to exist between the concentration of lead ions and aggregation of gold nanoparticles in the contact with the lead ions. The colorimetric detection of aqueous Pb^2+^ was performed at room temperature.

**Figure 7:**
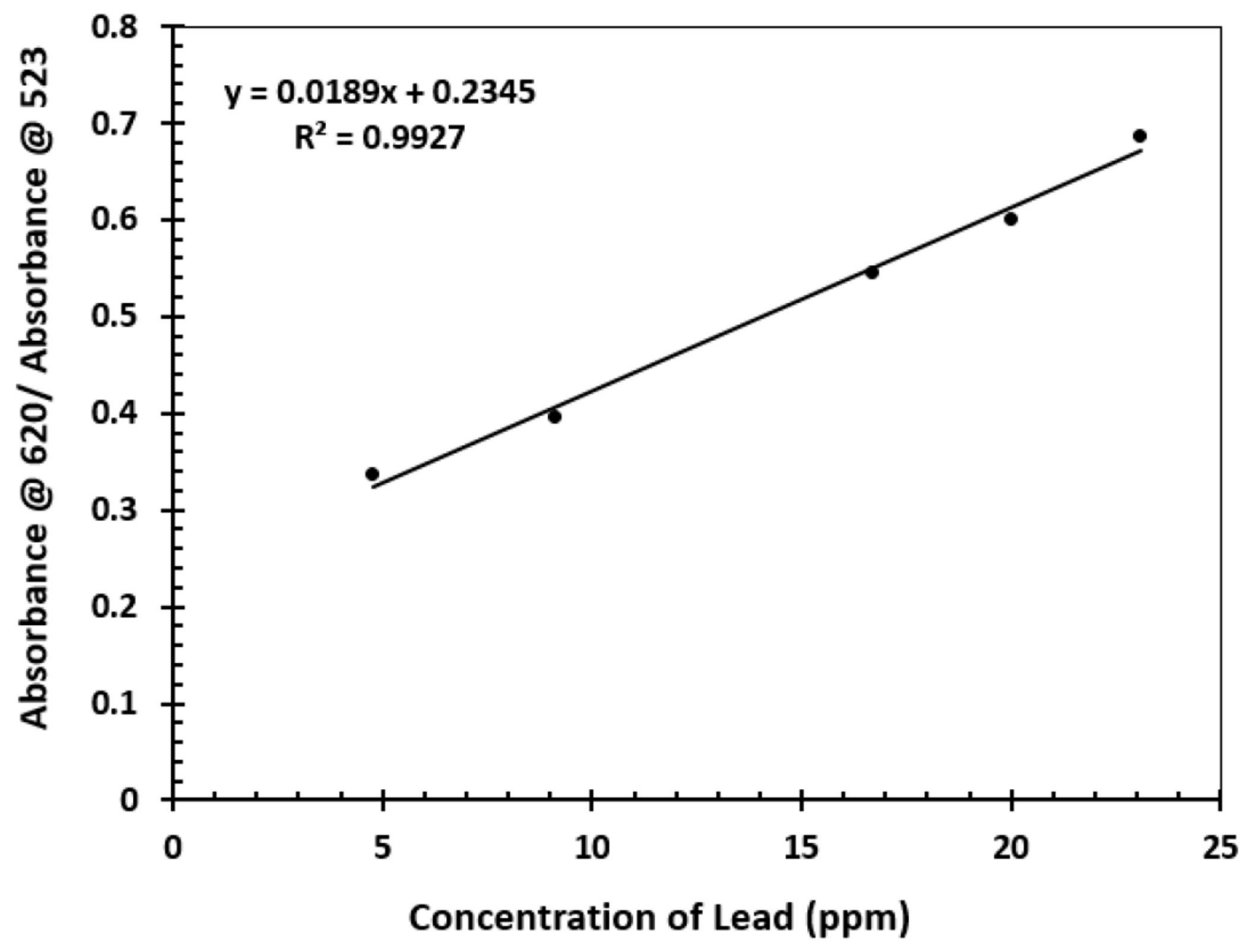
Calibration Curve for absorbance versus concentration of lead ions.

From the data of the UV-Vis, a curve of absorbance at 620 nm over the absorbance at 523 nm were computed and plotted over the concentration of lead in the solution. The correlation coefficient of this curve was 0.9927 as displayed in Figure 7. The calibration was then used to determine the concentration of Pb in water samples. At lower concentration of the lead the change in absorbance at 523 nm and 620 nm is low but at a higher concentration of lead, this change becomes more drastic. The curve is therefore linear at a higher concentration of lead than at a lower concentration of the same.

### Atomic absorption spectrometry

**Figure 8:**
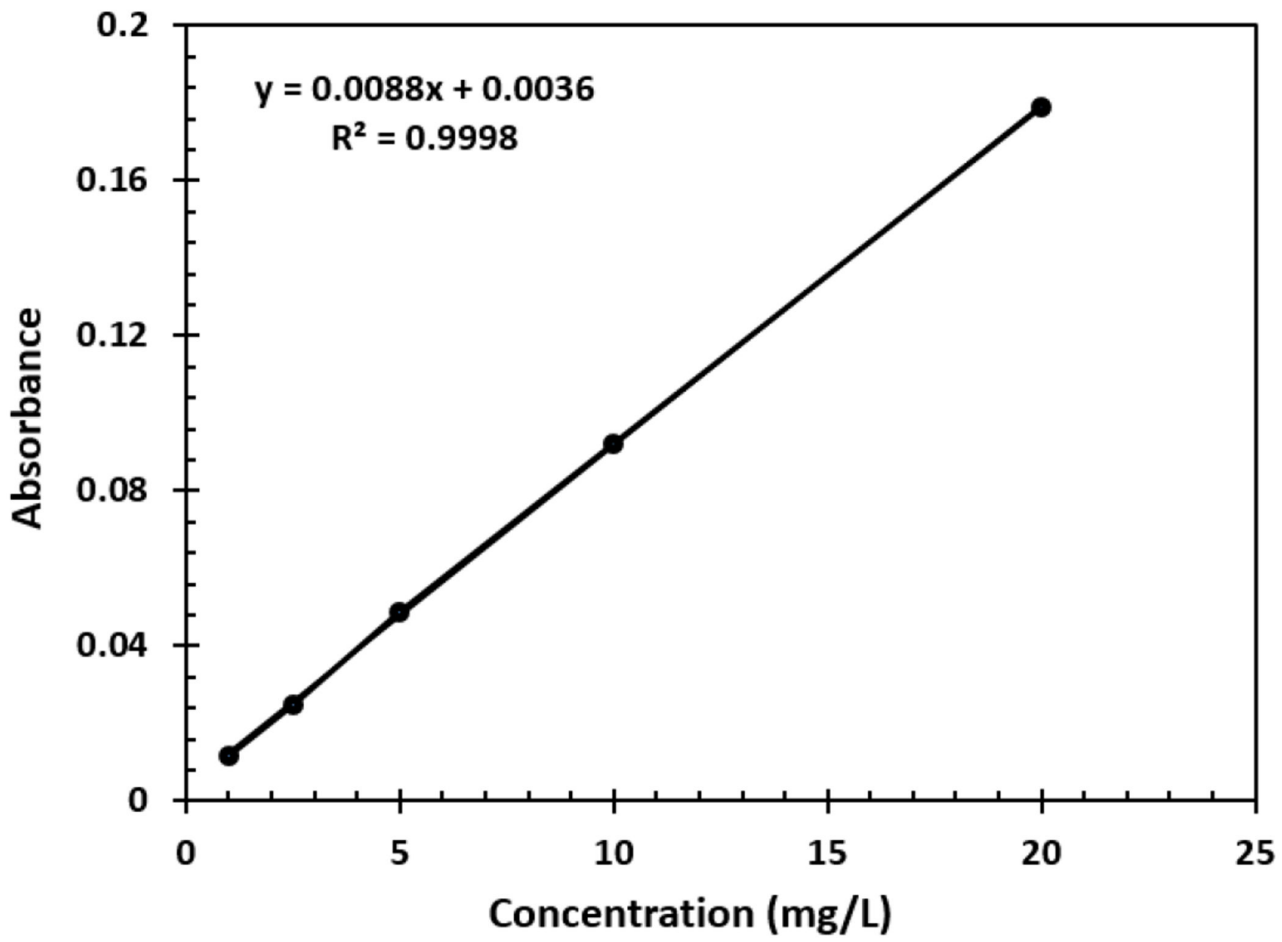
The calibration curve for atomic absorption analysis of lead.

Atomic Absorption Spectrometric measurements were also carried out to compare the results obtained by the gold nanoparticle detection method. Standards were prepared and used in the construction of the calibration curve for the atomic absorption measurements. Various solutions with different concentrations of lead, ranging from 1 to 25 ppm, were prepared. In all the solutions, nitric acid was added to enhance the amount of lead available for measurement. The correlation coefficient of the curve was 0.9998 as displayed in Figure 8. Next, water samples with varying amounts of lead were also measured using the calibration curve and the absorbance was correlated to the graph to determine the respective concentration. This data was then compared to that of the UV-Vis method employing the gold nanoparticle probe.

### Analysis of lead in watersheds

The colorimetric detection method was applied in the determination of lead in water samples drawn from liberty watershed, one of the three sheds that supply drinking water for the city of Baltimore (MD). Samples were taken over three different seasons (Summer 2018, Fall 2018 and Spring 2019) to see if Pb concentration changes with the seasons. Following the recommended protocol for sample preparation, Pb concentration was measured using AAS. It was observed that Pb^2+^ions detected in the water samples were negligible. To confirm the application of our colorimetric method, the samples were spiked with Pb^2+^ ions standard and tested. The concentration of Pb^2+^ ions in the water samples was 24 ppm, 12 ppm, and 4 ppm, respectively. All the measurements were conducted in triplicate and the values shown are averages. The results obtained for both the colorimetric testing with the gold nanoparticles using UV-Vis produced similar results as displayed in Table 1, indicating that the colorimetric detection method is suitable for testing lead in water bodies.

## CONCLUSION

Gold nanoparticles capped with lipoic acid were prepared using the Frens method of synthesis followed by ligand exchange reaction to replace citrate molecules with lipoic acid. The prepared particles were characterized using UV-Vis spectrometry, and Dynamic Light Scattering. The gold nanoparticle capped-lipoic acid was subsequently developed into a sensor capable of detecting lead ion in water. The lead ions interacted with the lipoic acid in solution which resulted in the aggregation of the particles to the degree of the concentration of the lead in solution. The morphology of the gold nanoparticles upon interaction with the lead was also examined using transmission electron microscopy. The results from Atomic Absorption Spectrometry corroborated with that of the gold nanoparticle sensor. The correlation coefficient of the calibration of the colorimetric method was 0.9927. The method was successfully used to determine the lead concentration of water samples collected from Tiberty watershed (one of the three sheds that supply drinking water for the city of Baltimore) over three seasons. The method thus provides a quick alternative technique for the detection and quantification of lead in water bodies and to the best of knowledge, constitutes a new colorimetric method for lead detection.

## Figures and Tables

**Table 1:** Determination of Pb ions in three different watersheds using both the colorimetric method and the AAS method.

Water samples	Concentration of Pb^2+^ (ppm)	
	Colorimetric method	AAS method
Summer 2018 #2	4.17 ± 0.25	4.27 ± 0.06
Fall 2018 #2	10.65 ± 1.13	11.16 ± 0.91
Spring 2019 #2	24.03 ± 1.0	24.37 ± 0.50

## References

[1] Elahi N, Kamali M, Baghersad MH. Recent biomedical applications of gold nanoparticles: A review. Talanta 2018;184:537–556. 29674080 10.1016/j.talanta.2018.02.088

[2] Huang X, El-Sayed MA. Gold nanoparticles: Optical properties and implementations in cancer diagnosis and photothermal therapy. Journal of Advanced Research. 2001 1(1)13–28.

[3] Dykman L, Khlebtsov N. Gold nanoparticles in biomedical applications: recent advances and perspectives. Chem Soc Rev 2012;41:2256–2282. 22130549 10.1039/c1cs15166e

[4] Athukorale S, De Silva M, LaCour A, Perera GS, Pittman CU, Zhang D, NaHS induces complete nondestructive ligand displacement from aggregated gold nanoparticles. J Phys Chem C 2018; 122:2137–2144.

[5] Zhou X, Xu W, Liu G, Panda D, Chen P. Size-dependent catalytic activity and dynamics of gold nanoparticles at the single-molecule level. J Am Chem Soc 2010;132:138–146. 19968305 10.1021/ja904307n

[6] Saha K, Agasti SS, Kim C, Li X, Rotello VM. Gold Nanoparticles in Chemical and Biological Sensing. Chem Rev 2012; 112(5):2739–2779. 22295941 10.1021/cr2001178 PMC4102386

[7] Dreaden EC, Austin LA, Mackey MA, El-Sayed MA. Size matters: gold nanoparticles in targeted cancer drug delivery. TherDeliv 2012;3(4):457–478. 10.4155/tde.12.21 PMC3596176 22834077

[8] Cole LE, Ross RD, Tilley JMR, Vargo-Gogola T, Roeder RK. Gold nanoparticles as contrast agents in x-ray imaging and computed tomography. Nanomedicine, 2015;10(2):321–341. 25600973 10.2217/nnm.14.171

[9] Wang D, Recent Advances in SPR Imaging Sensors. Sensors, 2019;19:12–66.

[10] El-Sayed MA. Some Interesting Properties of Metals Confined in Time and Nanometer Space of Different Shapes. Acc Chem Res 2001;34:257–264. 11308299 10.1021/ar960016n

[11] Zheng T, Bott S, Huo Q. Techniques for Accurate Sizing of Gold Nanoparticles Using Dynamic Light Scattering with Particular Application to Chemical and Biological Sensing Based on Aggregate Formation. ACS App Mat Inter 2016; 8(33): 21585–21594. 10.1021/acsami.6b06903 27472008

[12] Liu J, Cao Z, Lu Y. Functional nucleic acid sensors. Chem Rev 2009;109:1948–1998. 19301873 10.1021/cr030183i PMC2681788

[13] Slocik JM, Zabinski JS, Phillips DM, Naik RR. Colorimetric response of peptide-functionalized gold nanoparticles to metal ions. Small, 2008;4:548–551. 18383577 10.1002/smll.200700920

[14] Si S, Raula M, Paira TK, Mandal TK. Reversible self-assembly of carboxylated peptide-functionalized gold nanoparticles driven by metal-ion coordination. Chem Phys Chem 2008;9:1578–1584. 18615416 10.1002/cphc.200800121

[15] Hutter E, Pileni MPJ. Detection of DNA Hybridization by Gold Nanoparticle Enhanced Transmission SPR Spectroscopy. Phys Chem B 2003:107:6497–6499.

[16] Lee JS, Han MS, Mirkin CA. Colorimetric detection of mercuric ion (Hg2+) in aqueous media using DNA-functionalized gold nanoparticles. Chem Int Ed 2007;46:4093–4096. 10.1002/anie.200700269 17461429

[17] Liu CW, Huang CC, Chang HT. Control over surface DNA density on gold nanoparticles allows selective and sensitive detection of mercury (II). Langmuir 2008;24:8346–8350. 18582003 10.1021/la800589m

[18] Chai F, Wang CG, Wang TT, Ma ZF, Su ZM. Fluorescent Gold Nanoprobes for the Sensitive and Selective Detection for Hg. Nanotechnology. 2010;21:025501. 21124635 10.1007/s11671-010-9730-y PMC2964487

[19] Orr SE, Bridges CC. Chronic Kidney Disease and Exposure to Nephrotoxic Metals. Int J Mol Sci 2017; 18(5): 1039. 10.3390/ijms18051039 PMC5454951 28498320

[20] Canfield RL, Jusko TA, Kordas K. Environmental lead exposure and children’s cognitive function. Riv Ital Pediatr 2005;31(6):293–300. 26660292 PMC4675165

[21] Shen L, Chen Z, Li Y, He S, Xie S, Xu X, Electrochemical DNAzyme Sensor for Lead Based on Amplification of DNA-Au Bio-Bar Codes. Anal Chem 2008;80:6323–6328. 18627134 10.1021/ac800601y

[22] Zuo P, Yin BC, Ye BC. DNAzyme-based microarray for highly sensitive determination of metal ions Biosens. Bioelectron 2009;25:935–939. 10.1016/j.bios.2009.08.024 19740645

[23] Li T, Wang EK, Dong SJJ. Potassium-Lead-Switched G-Quadruplexes: A New Class of DNA Logic Gates. Am Chem Soc 2009; 131:15082–15083. 10.1021/ja9051075 19919152

[24] Zhou G, Chang J, Cui S, Pu H, Wen Z, Chen J, Real-Time, Selective Detection of Pb2+ in Water Using a Reduced Graphene Oxide/Gold Nanoparticle Field-Effect Transistor Device. ACS Appl Mat Inter 2014; 6(21): 19235–19241. 10.1021/am505275a 25296985

[25] Chen L, Li J, Chen L. Colorimetric Detection of Mercury Species Based on Functionalized Gold Nanoparticles. ACS Appl Mat Inter 2014;6(18): 15897–15904. 10.1021/am503531c 25153162

[26] Sang F, Liu J, Zhang X, Pan J. An aptamer-based colorimetric Pt(II) assay based on the use of gold nanoparticles and a cationic polymer. Microchimica Acta 2018; 185(5). 10.1007/s00604-018-2794-6 29696378

[27] Ratnarathorn N, Chailapakul O, Dungchai W. Highly sensitive colorimetric detection of lead using maleic acid functionalized gold nanoparticles. Talanta 2015;132:613–618. 25476352 10.1016/j.talanta.2014.10.024

[28] Xia H, Xiahou Y, Zhang P, Ding W, Wang D. Revitalizing the Frens Method To Synthesize Uniform, Quasi-Spherical Gold Nanoparticles with Deliberately Regulated Sizes from 2 to 330 nm. Langmuir 2016;32(23):5870–5880. 27263542 10.1021/acs.langmuir.6b01312

[29] Zabetakis K, Ghann WE, Kumar S, Daniel MC. Effect of high gold salt concentrations on the size and polydispersity of gold nanoparticles prepared by an extended Turkevich-Frens method. Gold Bulletin 2012;45(4): 203–211.

[30] Daniel MC, Astruc D. Gold Nanoparticles: Assembly, Supramolecular Chemistry, Quantum-Size-Related Properties, and Applications toward Biology, Catalysis, and Nanotechnology. Chem Rev 2004;104(1):293–346. 14719978 10.1021/cr030698+

